# 

**Published:** 1888-02-04

**Authors:** 


### SPECIAL NOTICE TO NEWSAGENTS, ADVERTISERS, AND THE TRADE.

### CHANGE OF OFFICE AND PUBLISHER.

Notice is hereby given, that the Office of The Hospital will henceforth be at 38, Old Jewry (Cheapside end), E.C., to which address all communications should be sent. Mr. A. Doig has been appointed Advertising Agent, and Mr. J. H. S. Hanning, Manager and Publisher. All Cheques and Post Office Orders should be made payable to J. H. S. Hanning, 38, Old Jewry, E.C., crossed Lloyds, Barnetts, and Bosanquets Bank (Limited).

### QUESTIONS AND ANSWERS.

### "HOSPITALS FOR INCURABLES."

SENEX writes: "Will anyone tell me whether there is any hospital for incurables which admits a patient by the subscribers or governor's letter without payment and without collecting votes?"

*** The following institutions, we believe, fulfil the conditions included in Senex's letter: Border Counties Home for Incurables, Stanwix, Carlisle; the Midland Counties Home and Hospital for Chronic and Incurable Diseases, Leamington (except that payment is required in each case of from 7*s.* to 10*s.* 6*d.* per week according to the means of the patient). The only institution which admits these cases free by the selection of the committee, so far as we are aware, is the Hospital of St. John of Jerusalem and St. Elizabeth of Hungary, 47, Great Ormond Street, London, W.C. We should be glad of information on this point from any of our readers.

A SINGLE drop of nicotine applied to the eye of a cat will kill the animal in a few minutes; a small bird will die from the inhalation of a portion of nicotine vapour so small as to be inappreciable by a fine balance. Rabbits, cats, and dogs die in from twenty to thirty seconds with less than a drop placed on the tongue, so rapidly is it absorbed, and so violent are its effects; and man suffers severely with only the twenty-fifth of a grain.

### Amusements with Profit for all Ages.

**Rules.**—All answers must be addressed to the Prize Editor, The Hospital, 38, Old Jewry, Cheapside, London, E.C., not later than the *first* post on Thursday next. The full name and address must in all cases be given, but a *nom de plume* may be added for publication. Only one side of the paper must be written on.

### FOR MEN AND WOMEN.

### Acrostic Competition.

Commenced November 26th, 1887; ends February 18th, 1888.

A PRIZE OF ONE GUINEA will be given to the competitor who gains the highest number of marks for best original acrostics during the ensuing quarter. All acrostics sent in for this competition will be credited according to merit, twelve marks being the highest score given for one acrostic.

A PRIZE of HALF-A-GUINEA to be given to the competitor who gains the highest score for solution of acrostics set, including the one inserted on November 26th. One mark only will be given to the competitors who solve all the lights of each acrostic.

These competitions to alternate weekly.

This week credit will be given for Solution of the following:—

No. 6. Double Acrostic.
"The First Day."

1. "Fade with thy bowers, O land of visions, fade!
From thee no voice came o'er the gloomy deep
To bid man cease to weep."
2. "The roar of waters! from the headlong height,
The flashing mass foams, shaking the abyss."
3. "She who knew that love could vanquish death,
Who, kneeling with one arm around her king,
Drew forth the poison with her balmy breath,
Sweet as young buds in spring."
4. "To him, as to the burning levin,
Short bright resistless course was given,
Till burst the bolt on yonder shore,
Rolled, blazed, destroyed, and was no more."
5. "So our wandering band shuns all warning,
And in every soil plants its hold,
Each tract of Old England adorning
With the folds of the Red, Black, and Gold."
6. "I have warred with a world which vanquished me only
When the meteor of conquest allured me too far;
I have coped with the nations which dread me thus lonely,
The last single captive to millions in war."
7. "Now came still evening on, and twilight grey
Had in her sober livery all things clad."

The author of the quotations must be given.

### Answers.

No. 5. Double Acrostic.

O per A

R ebe C

I ntege R

G alile O

I ri S

N ough T

A lib I

L ogi C

Correct answers have been received from Lady Clara, Esau, Pasteaur, Reldas, Brownie, Mater, Aralc, Canice, Condorcet, Dunce, E. E.

### II. Word and Literary Competition.

Second Quarterly Competition commences February 4th, 1888; ends April 28th, 1888.

Three prizes of one guinea, 15*s.* and 10*s.* 6*d.* will be given each quarter to the three competitors who obtain the highest number of marks: (1) by making the largest number of words derived from the word or phrase set for anatomical dissection; or (2) for the best answers to (6); or (3) by (1) and (2) combined.

(A) THE WORD COMPETITION.

We shall give the newspapers and periodicals for dissection during this quarter, that for this, the *first* week of the quarter, being

"Advertiser."

Observe.—Proper names, abbreviations, foreign words, words of less than four letters and repetitions are barred. Nuttall's *Standard* dictionary only to be used.

### Results of Twelfth Week.

*The Field.*—Ellice (60), Gretta (58), Reldas (56), E. E. (47), K. M. (46), Lauriston (39), Tabs (34), Condorcet (31).

(B) THE LITERARY COMPETITION.

Some people hate puzzles of all kinds, including word competitions, but they are fond of correspondence, and many sisters and mothers are continually receiving and sending letters to and from members of the family who are abroad in India or the Colonies. The constant coming and going between England and other portions of the British Empire make it a matter of interest and importance to know something about the life, the climate, the amusements, the adventures, the clothing, and the surroundings of those who seek a livelihood beyond the seas. We shall therefore try to find space for the publication of selected letters from abroad, which may be sent by any of our readers who have friends and relations in foreign lands. We shall give marks for these letters as well as for the word competitions which will count equally for these prizes, so that anyone may enter for both or either, the prizes to be given to those who get the highest number of marks.

Gretta sends a letter from Calcutta this week.

*Results of the first quarterly word and literary competition will be published next week.*

### Letter from Gibraltar.

The following letter may be of interest to those persons who, like the writer, are compelled by delicate health to seek a warmer climate for the winter:

I am going to keep this letter open for a few days, and I shall write of anything that I think will interest you as it occurs to me. In the first place it is wonderfully cheap. Meat is only 7d. a lb., turkeys 6d., and nice chickens 1s. 6d. each. Fruit, vegetables, and flowers are to be bought for almost nothing, and first-rate wine costs 1s. a bottle. I drink goat's milk, which I find most nourishing, twice a day, in addition to the wine which I am ordered, and I am growing quite stout in consequence of all the good things I am able to take. To-day is the 2nd of January, and I am writing with windows wide open and no fires. I wish you were here. The journey is the only expense. My rooms are very comfortable, and I have been very fortunate in meeting with an English landlady, which is rare here. She is so nice and kind; her servant is Spanish. The organist of the Protestant church and his wife have rooms next to mine, and I often have some nice music. Yesterday I drove to the end of the Rock, a lovely drive. Everything so green and fresh, for we have had a good deal of rain lately. There were quantities of scarlet cactus and geraniums all along the road; the orange trees were covered with fruit, and the wild narcissus scenting the air with its fragrance, combined to make it a perfect summer day. I think the Spanish language is very pretty and soft. There are all sorts and conditions of men to be seen in the streets here, in an endless variety of costumes: the Moors, who dress according to their rank; soldiers and sailors of different nationalities; monks and priests with their curious hats and cloaks: and nuns and ladies in their mantillas. It is odd to see turkeys driven in flocks like sheep, and donkeys laden with all sorts of things. The Spanish music is very strange and weird, and the church bells have the saddest and weirdest sound. There is a library here, to which I belong. You have to be proposed and elected as a member. There are ladies and gentlemen's rooms, comfortable lounging chairs, and all the papers, &c. There are nice gardens attached, and one can write one's letters at the club, read, and meet one's friends; so that I find it a very pleasant rendezvous. I hope to be home at the end of March.

### The Work Distribution.

Our apologies are due to Miss M. A. Shone (Canice) for accidentally omitting to announce last week that her very handsome and valuable doll, representing a Norwegian peasant, has been sent to the Children's Hospital, Paddington Green. The matron was so pleased with this charming present that she kept it in clover for the special amusement of the little ones, who come to her room during a portion of most days. "Canice" should call at the hospital and see how much enjoyment her splendid doll has given to the children.

### THE CHILDREN'S CORNER.

PRIZES.
FOR MOST CORRECT ANSWERS TO PUZZLES.

This Commenced October 1st, 1887.

One Pound a Month.

A PRIZE OF THREE GUINEAS

to the Competitor who shall have sent in the largest number of correct or best answers during the twelve months.

### Puzzles—First Week.

No. 1 Historical Charade.—My *first* is a vessel which when full cannot receive my *second.* My whole is a name given to Malcolm, king of Scotland.

No. 2. Dessert Enigmatically Expressed.—1. Two-thirds of an animal, two-fourths of a verb and a house. 2. Dutch princes. 3. To cut. 4. A tree and a fruit.

No. 3. Transposition.

Complete, you'll find I'm a substance hard,
Yet oft of genuine worth and high regard;
*Behead* me, I become a dulcet sound,
Which, if *transposed*, will still alike be fonnd;
*Reverse* me, I conduct you to a college
For learning famed, and every classic knowledge.

No. 4. Find what 100 + a + 50 + 1 + 100 + o =a very useful article.

### Results of Third Week Puzzles.

No. 9 Geographical Charade.—Cash-mere =Cashmere.

No. 10. Diamond Puzzle.

N
B O W
T U R I N
N O R W I C H
S E I N E
I C I
H

No. 11. Enigma.—Caper.

No. 12. Biographical Anagrams.—Bonaparte, William Tell.

*All right*—Spider, Excelsior, Toad, Scrooge, Tim. *Three right*—A. C. K., Plummy Fluff, Druce, Jacko, P. S., Gem, A. G. S., McCulloch. *Two right*—Happy Eliza, Robinson, Fern, Judy.

### Notices to Correspondents.

*Brisbane.*—Answers received too late.

*A.C.K., Druce.*—I regret that *no exception* to the general rules can be made.

All competitors who send in papers must strictly abide by the following

**Conditions.**

I. Every paper sent in must be clearly written, and one side only must be used. All competitors must mark at the head of their papers the number of their competition.

II. Each paper must be signed by the author with his or her real name and address. A *nom de plume* may be added, if the writer does not desire to be referred to by us by his real name. In the case of all prize-winners, however, the real name and address will be published.

III. All communications relating to prize competitions should be addressed to "The Prize Editor, The Hospital, 38, Old Jewry, London, E.C." Competitors are requested not to include in letters, etc., to the Prize Editor communications on matters of other business. All letters must be fully prepaid.

IV. The Prize Editor of "The Hospital" is the sole judge of the competitions, and his decision is in all cases final and without appeal.

V. The Prize Editor reserves the right to publish any paper sent in, whether it receives a prize or not. He does not hold himself responsible for any MSS. sent, nor can he undertake to return rejected competitions.

**Notice to all Correspondents.**—N.B.—All letters referring to this page, which do not arrive at 38, Old Jewry, Cheapside, London, E.C., by the *first post on Thursdays,* and are not addressed PRIZE EDITOR, will in future be disqualified and disregarded.

No one will be permitted to win the Monthly or Quarterly Prizes twice in any one year, the annual prizes being offered especially to prevent this.

